# Integrating non-communicable disease screening to COVID-19 and human immunodeficiency virus care in Johannesburg South Africa: a pilot model for low- and middle-income countries

**DOI:** 10.3389/fpubh.2026.1825868

**Published:** 2026-09-17

**Authors:** Niraksha Jithoo, Nomathemba Chandiwana, Oludoyinmola Ojifinni, Jeffery Wing

**Affiliations:** 1 Viatris Global NCD COE, Johannesburg, South Africa; 2 Desmond Tutu HIV Centre, University of Cape Town, Cape Town, South Africa; 3 Wits Ezintsha, University of the Witwatersrand, Johannesburg, South Africa; 4 School of Clinical Medicine, Faculty of Health Sciences, University of the Witwatersrand, Johannesburg, South Africa; 5 Division of Endocrinology, Department of Internal Medicine, Faculty of Health Sciences, University of the Witwatersrand, Johannesburg, South Africa; 6 Donald Gordon Medical Centre, Johannesburg, South Africa

**Keywords:** health care, HIV, integrated care, NCDS, South Africa

## Abstract

**Background:**

The COVID-19 pandemic underscored the relationship between infectious diseases and non-communicable diseases (NCDs), highlighting increased risks for people with pre-existing chronic conditions. This study piloted an integrated care model for NCD and infectious disease screening in healthcare settings. The aim of this study is to show that integrating NCD screening into existing HIV clinics and COVID-19 vaccine delivery clinics leads to better pandemic preparedness.

**Methods:**

From October 2023 to December 2023, we conducted a cross sectional prospective, low interventional study involving 461 participants from two health care clinics in Johannesburg, South Africa using convenience sampling. Screening included blood pressure, body mass index (BMI), HbA1c, lipid profile, glucose, and kidney function assessments. Data were analyzed to identify NCD prevalence and key risk factors.

**Results:**

Newly diagnosed NCDs were found in 73.9% of participants. The most common were kidney disease (31.7%), obesity (28.9%), and dyslipidaemia (25.8%). Modifiable cardiovascular disease (CVD) risk factors, including obesity (63.9%) and alcohol use (50%), were widespread.

**Conclusion:**

Integrating NCD screening into HIV and COVID-19 services is achievable, offers a valuable opportunity for early detection and intervention. Scaling such models could strengthen health systems in resource-limited settings.

## Introduction

The COVID-19 pandemic brought renewed attention to the often-overlooked link between infectious diseases and NCDs (1). Individuals with NCDs faced higher risks of severe outcomes and mortality from SARS-CoV-2, underscoring the biological and health system vulnerabilities created by multimorbidity (2–4). These risks were most acute in low- and middle-income countries (LMICs), where weak primary care systems and siloed disease programmes failed to meet the needs of patients with complex chronic conditions (1, 5, 6).

Globally, NCDs account for 41 million deaths annually, with more than 80% occurring in LMICs (7). In South Africa, NCDs now account for over half of all deaths (57.4%), with CVD, T2D, and chronic respiratory conditions leading the burden (8). Despite global momentum behind cost-effective “best buy” interventions to help countries meet NCD targets under Sustainable Development Goal (SDG.) 3.4, implementation has been uneven (9). Progress has been made, particularly in taxation of tobacco and alcohol, however, efforts to improve diets through salt and sugar intake reduction or elimination of trans-fats remain slow. As a result, current efforts fall short of achieving SDG goal of reducing premature deaths by 33% by 2030 (9).

South Africa has a quadruple burden of disease namely communicable diseases such as HIV and tuberculosis (TB), maternal and child morbidity mortality, violent crimes as well as a rising NCD prevalence (10). Chronic kidney disease (CKD), often linked to unmanaged hypertension (HPT) and diabetes, represents a growing but under-recognised threat.

The pandemic not only amplified clinical risks for individuals with NCDs but also disrupted essential health services. World Health Organization’s (2) national pulse survey reported that 90% of countries experienced interruptions to NCD care, with the largest gaps in HPT, diabetes, and mental health services. In South Africa, excess mortality during the pandemic was disproportionately driven by people with poorly managed chronic diseases. These disruptions exposed structural weaknesses in health systems but also created new opportunities for innovation.

Integrated service delivery models, leveraging existing platforms such as HIV and COVID-19 programmes, offer a pragmatic and cost-efficient pathway to strengthen chronic care. Evidence from trials such as INTE-AFRICA and recent work by the NCD Alliance demonstrate that integrated care not only improves health outcomes but also reduces patient costs and improves access (10–13). However, real-world implementation remains limited, and few studies have examined feasibility within urban primary care settings in southern Africa.

In this pilot study, we evaluate the implementation of an integrated NCD screening model within two healthcare clinics in Johannesburg, South Africa. Building on COVID-19 and HIV infrastructure we aimed to identify the prevalence of HPT, obesity, T2D, dyslipidaemia, and CKD, while also assessing cardiovascular risk profiles and training primary healthcare staff in referral protocols. Our findings aim to inform scalable models for integrated chronic care in urban African settings.

The overall objective was to leverage COVID-19 and HIV pandemic response infrastructure for screening and monitoring of NCDs using NCD risk assessment tools.

The primary objective of the study was to assess the prevalence, incidence, and impact of CVD risk factors in HPT, obesity, T2D, dyslipidemia patients receiving COVID-19 vaccines or ART.

The secondary objective was:

Integrated care: training and supervision improvement of nurses to establish a primary health care (PHC) model of screening, and management, referral, and follow-up for HPT, T2D, dyslipidemia patients receiving COVID-19 vaccines or ART. Thus, facilitating integration of NCDs and HIV management.

## Methods

### Study design and sites

This cross sectional prospective, low interventional, pilot feasibility study was conducted, from October 2023 to December 2023 at two health care clinics in Johannesburg, South Africa. The first site, Riverpark Clinic in Alexandra Township is a large site, that provides nurse-led chronic disease management, COVID-19 vaccination and other primary care services. The second site, Ezintsha, Clinical Trials Unit, is a research unit of the University of the Witwatersrand that focuses on HIV care and clinical research, with advanced diagnostic capabilities for urban populations. Both sites provide services to underserved populations, living in urban, crowded and poorer communities of Johannesburg. The study was approved by Human Resource Ethics Committee.

### Participants

Adults aged 18 and older attending either clinic for antiretroviral therapy (ART) or COVID-19 vaccinations were eligible. Informed consent was obtained from all participants. Participants were approached while queuing and invited to join the study without losing their place in line. Convenience sampling was used. As a pilot feasibility study, a formal prior sample size or power calculation was not performed. The sample size was determined by the duration of the study period and practical clinic constraint. Informed consent was obtained before any procedures. Participants were excluded if they were unable or unwilling to provide consent, or unable to complete all study procedures. Additionally, individuals judged by investigators to be at significant risk of protocol noncompliance were excluded.

### Measures

We used standardised protocols to collect anthropometric and clinical data. These included height, weight, and body mass index (BMI), calculated as weight (kg) divided by height squared (m^2^). Blood pressure and pulse were measured to screen for HPT, defined according to South African HPT Society guidelines. Point-of-care (POC) tests assessed HbA1c, total cholesterol, HDL, random plasma glucose, serum creatinine, estimated glomerular filtration (eGFR) and urine microalbumin levels. Definitions and clinical thresholds are detailed in Appendix 1.

### Point-of-care testing equipment

POC testing was conducted using the Cobas b 101 system (Roche Diagnostics), a compact, portable analyser suitable for multi-analyte testing from capillary blood samples. This device allowed rapid results for HbA1c, lipids, and glucose making it ideal for resource-limited settings. Urine microalbumin was measured using the Clinitek Microalbumin Reagent Strips with a handheld analyser.

## Statistical analysis

Data from 460 participants with complete records were exported from REDCap and analysed using SPSS Version 29. Bivariate analyses were conducted to identify associations at a 5% significance level. Primary outcomes included the prevalence of NCDs (HPT, T2D, obesity, dyslipidaemia, and kidney impairment). Cardiovascular risk was estimated using the Framingham risk score. Kidney disease classification followed KDIGO CKD 2024 guidelines. Variables with *p*-values ≤ 0.05 were included in logistic regression models to assess predictors of kidney impairment.

### Sociodemographic and clinical characteristics

A total of 460 participants had complete information and were included in the analysis. Table 1 shows the male to female comparison of participants’ sociodemographic and clinical characteristics. Most of the study participants were female (74.8%), black/African (99.1%) and 50.5% were unemployed. The mean BMI was significantly higher at 34.56 ± 7.67 among the female compared with 27.02 ± 5.92 among the male participants (*p* < 0.001). There was also significant male to female differences in the family history of NCDs and substance use among the study participants. The mean systolic blood pressure was 135.64 ± 21.38 mmHg while the mean diastolic blood pressure was 85.01 ± 13.45 mmHg. About one-third (31.3%) of the participants had blood pressure measurements consistent with a diagnosis of HPT. Of these, 5.6% had isolated systolic HPT. Only 25 (5.4%) of the participants had random plasma glucose >11.0 mmol/L while 26.1% were classified as diabetic based on the HbA1c results. Of the 120 participants with HbA1c ≥ 6.5%, 93 had a pre-existing diagnosis of diabetes mellitus and were already on treatment. The remaining 27 participants represented newly detected cases with no prior diagnosis.

**Table 1:** Sociodemographic and clinical characteristics.

Variable	Female	Male	Total^$^	Chi square	*p* value
Study location					
Ezintsha	154 (70.0)	66 (30.0)	220 (100)	5.114	0.025*
Riverpark	190 (79.2)	50 (20.8)	240 (100)		
Age					
19 to 29	38 (76.0)	12 (24.0)	50 (100)	6.203	0.184
30 to 39	106 (81.5)	24 (18.5)	130 (100)		
40 to 49	100 (69.9)	43 (30.1)	143 (100)		
50 to 59	58 (76.3)	18 (23.7)	76 (100)		
≥ 60	42 (68.9)	19 (31.1)	61 (100)		
Mean age	43.90 ± 12.22	45.39 ± 12.69			
Race					
Black/African	340 (74.6)	116 (25.4)	456 (100)	1.361^#^	0.715
White	2 (100)	0 (0.0)	2 (100)		
Coloured	1 (100)	0 (0.0)	1 (100)		
Indian/Asian	1 (100)	0 (0.0)	1 (100)		
Employment status^$^					
Employed	162 (73.6)	58 (26.4)	220 (100)	0.326	0.589
Unemployed	177 (76.0)	56 (24.0)	233 (100)		
Covid-19 vaccination^$^					
Yes	275 (77.9)	78 (22.1)	353 (100)	7.213	0.008*
No	69 (65.1)	37 (34.9)	106 (100)		
BMI					
Normal weight (18.5–24.9)	33 (38.8)	52 (61.2)	85 (100)	89.926	<0.001*
Pre-obesity (25.0–29.9)	56 (69.1)	25 (30.9)	81 (100)		
Obesity class I (30.0–34.9)	116 (81.1)	27 (18.9)	143 (100)		
Obesity class II (35.0–39.9)	66 (85.7)	11 (14.3)	77 (100)		
Obesity class III (≥ 40)	73 (98.6)	1 (1.4)	74 (100)		
Mean BMI	34.56 ± 7.67	27.02 ± 5.92			
Family history of NCDs^$^					
Yes	187 (82.7)	39 (17.3)	226 (100)	16.200	<0.001*
No	149 (66.2)	76 (33.8)	225 (100)		
Tobacco use					
Yes	50 (56.2)	39 (43.8)	89 (100)	20.250	<0.001*
No	294 (79.2)	77 (20.8)	371 (100)		
Smoking status^$^					
Non-smoker	294 (79.2)	77 (20.8)	317 (100)	40.865	<0.001*
Current smoker	31 (44.9)	38 (55.1)	69 (100)		
Other tobacco use	19 (95.0)	1 (5.0)	20 (100)		
Illicit drug use					
Yes	3 (37.5)	5 (62.5)	8 (100)	6.001	0.027*
No	341 (75.4)	111 (24.6)	452 (100)		
Drinks alcohol					
Yes	158 (66.9)	78 (33.1)	236 (100)	15.770	<0.001*
No	186 (83.0)	38 (17.0)	224 (100)		
Haemoglobin A1c (%)^$^					
< 6.5 (inconclusive)	248 (74.7)	84 (25.3)	332 (100)	0.183	0.713
≥ 6.5 (diabetic)	92 (76.7)	28 (23.3)	120(100)		
Estimated GFR category					
G1: Normal or high	240 (78.7)	65 (21.3)	305 (100)	14.686^#^	0.005*
G2: Mildly decreased	92 (70.8)	38 (29.2)	130 (100)		
G3a: Mildly to moderately decreased	8 (50.0)	8 (50.0)	16 (100)		
G3b: Moderately to severely decreased	4 (57.1)	3 (42.9)	7 (100)		
G4: Severely decreased	0 (0.0)	2 (100)	2 (100)		
Estimated GFR in two categories					
Normal or high GFR (G1)	240 (78.7)	65 (21.3)	305 (100)	7.323	0.007*
Decreased GFR (G2 to G4)	104 (67.1)	51 (32.9)	155 (100)		
Albumin-creatinine ratio$					
< 3 mg/mmol	330 (74.3)	114 (25.7)	444 (100)	1.170	0.279
3–30 mg/mmol	13 (86.7)	2 (13.3)	15 (100)		
Random plasma glucose (mmol/L)					
< 5.6 (diabetes excluded)	199 (72.6)	75 (27.4)	274 (100)	2.212	0.331
5.6–11.0 (inconclusive)	127 (78.9)	34 (21.1)	161 (100)		
≥ 11.1 (diabetic)	18 (72.0)	7 (28.0)	25 (100)		
Total cholesterol^$^					
< 200 mg/dL (desirable)	339 (74.8)	114 (25.2)	453 (100)	0.579^#^	0.447
200–239 mg/dL (borderline)	1 (100)	0 (0.0)	1 (100)		
HDL					
< 40 mg/dL (low – increased risk)	327 (75.3)	107 (24.7)	434 (100)	7.832	0.020*
40–59 mg/dL (borderline)	9 (56.3)	7 (43.8)	16 (100)		
≥ 60 mg/dL (high/protective)	9 (100)	0 (0.0)	9 (100)		
Blood pressure categories					
Normal	74 (80.4)	18 (19.6)	92 (100)	9.554^#^	0.145
Optimal blood pressure	28 (80.0)	7 (20.0)	35 (100)		
High normal blood pressure	138 (73.0)	51 (27.0)	189 (100)		
Grade 1 HPT	70 (79.5)	18 (20.5)	88 (100)		
Grade 2 HPT	20 (64.5)	11 (35.5)	31 (100)		
Grade 3 HPT	9 (52.9)	8 (47.1)	17 (100)		
Isolated systolic HPT	5 (62.5)	3 (37.5)	8 (100)		
Mean systolic blood pressure	134.32 ± 20.76	139.57 ± 22.74			
Mean diastolic blood pressure	82.51 ± 13.78	84.73 ± 12.35			
HPT					
Yes	104 (72.2)	40 (27.8)	144 (100)	0.729	0.418
No	240 (75.9)	76 (24.1)	316 (100)		
Newly diagnosed NCD					
Yes	251 (73.8)	89 (26.2)	340 (100)	0.636	0.465
No	93 (77.5)	27 (22.5)	120 (100)		
Newly diagnosed diabetes mellitus					
Yes	21 (77.8)	6 (22.2)	27 (100)	0.220	0.820
No	232 (73.7)	83 (26.3)	315 (100)		
Newly diagnosed HPT					
Yes	32 (61.5)	20 (38.5)	52 (100)	4.794	0.039*
No	219 (76.0)	69 (24.0)	288 (100)		
Newly diagnosed dyslipidaemia					
Yes	96 (80.7)	23 (19.3)	119 (100)	4.195	0.041*
No	155 (70.5)	65 (29.5)	220 (100)		
Newly diagnosed kidney disease					
Yes	105 (71.9)	41 (28.1)	146 (100)	0.520	0.533
No	147 (75.4)	48 (24.6)	195 (100)		
Framingham 10-year risk category					
Low risk	159 (88.8)	20 (11.2)	179 (100)	54.123	<0.001*
Medium risk	155 (70.8)	64 (29.2)	219 (100)		
High risk	26 (55.3)	21 (44.7)	47 (100)		
Very high risk	0 (0.0)	8 (100)	8 (100)		
Referred following screening					
Yes	265 (74.0)	93 (26.0)	358 (100)	0.123	0.726
No	72 (75.8)	23 (24.2)	95 (100)		

^#^Likelihood ratio reported because of cells with expected counts <5 *statistically significant. ^$^ Totals vary across variables due to missing data. Percentages are calculated using the valid *N* for each variable. There were some missing clinical data (e.g., employment status, family history, smoking history, newly diagnosed NCD’s) which were due to subjects “not knowing” or “refusing” to answer. For instance, many subjects are not formally employed, worked intermittently, rely on family support and/or social grants or a combination of these. The POC (HbA1c, Cholesterol, and Albuminuria) discrepancies were related to “device days off” when consumable shortages were experienced (wastage, re-calibration needs). We elected to include these patients in the analysis in line with the goal of “real world” challenges in this pilot study.

While 66% of the study population had normal to high glomerular filtration rates (GFR), the proportion who had impaired kidney function was significantly higher among the females than the male participants. The cardiovascular risk measured with the Framingham 10-year risk classification showed that 38.9% of the study population had low risk, 12% had high to very high risk while eight (8) men, i.e., 1.7% of the population had very high risk.

### HIV status and multimorbidity prevalence

While 17 (3.7%) of the population had no NCDs reported or diagnosed in the study, 11 (2.4%) of the participants had four NCDS while 69 (15.0%) had three (Table 2). The prevalence of multimorbidity in the study population was 17.4%. The proportion who had co-existing HIV and multimorbidity was 12.8%.

**Table 2:** HIV and co-existing NCD.

Variable	HIV positive	HIV negative	Total	Chi square	*p* value
Multimorbidity					
Yes	23 (12.8)	57 (21.3)	80	5.375	0.020
No	157 (87.2)	210 (78.7)	367		
Newly diagnosed NCDs					
Yes	163 (85.8)	177 (65.6)	340	23.680	<0.001
No	27 (14.2)	93 (34.4)	120		
Newly diagnosed diabetes					
Yes	15 (9.1)	12 (6.7)	27	0.679	0.410
No	149 (90.9)	166 (93.3)	315		
Newly diagnosed HPT					
Yes	16 (9.8)	147 (90.2)	52	7.253	0.007
No	36 (20.3)	141 (79.7)	288		
Newly diagnosed dyslipidaemia					
Yes	81 (50.0)	38 (21.5)	119	30.224	<0.001
No	81 (50.0)	139 (78.5)	177		
Newly diagnosed renal disease					
Yes	109 (66.9)	37 (20.8)	146	73.805	<0.001
No	54 (33.1)	141 (79.2)	178		

### Predictors of kidney impairment

We examined the factors associated with renal impairment assessed with the GFR (Table 3). Factors that were significant on bivariate analysis were fitted into a logistics regression model. We performed a binary logistic regression using the Enter method to assess the impact of study location, sex, age, BMI, family history of NCDs, smoking status, tobacco use, HIV status, blood glucose, use of antiretroviral and diabetes medications and the cardiovascular risk measure with the Framingham risk category on the likelihood of kidney impairment measured with the GFR. The Hosmer Lemeshow goodness of fit test showed that the model was a good fit (χ^2^ = 8.261 at 8 degrees of freedom with *p* value = 0.408). Our findings showed that females had a higher risk of kidney impairment than males. The risk of kidney impairment was also higher among people who were 45 years and over compared with those who were less than 35 years old. Other significant predictors of kidney impairment in this population were BMI, random plasma glucose in the diabetic range, having a newly diagnosed NCD and cardiovascular risk were significant predictors of kidney impairment in this study population.

**Table 3:** Predictors of kidney impairment.

Variable	Decreased GFR *n* (%)	Adjusted odds	95% CI
Study location			
Ezintsha	104 (47.3)	Ref	
Riverpark	51 (21.3)	0.852	0.422–1.720
Sex			
Female	104 (30.2)	2.855	1.392–5.855*
Male	51 (44.0)	Ref	
Age			
<35	21 (13.5)	Ref	
35–44	49 (31.6)	1.493	0.594–3.750
45–64	64 (41.3)	4.866	1.584–14.946*
≥ 65	21 (13.5)	19.011	3.152–114.667*
BMI			
Normal weight (18.5–24.9)	60 (70.6)	Ref	
Pre-obesity (25.0–29.9)	46 (56.8)	0.403	0.182–0.892*
Obesity class I (30.0–34.9)	34 (23.8)	0.093	0.040–0.211*
Obesity class II (35.0–39.9)	8 (10.4)	0.023	0.007–0.074*
Obesity class III (≥ 40)	7 (9.5)	0.023	0.007–0.074*
Family history of NCDs			
Yes	62 (27.4)	0.990	0.578–1.696
No	89 (39.6)	Ref	
Tobacco use			
Yes	40 (44.9)	0.777	0.215–2.814
No	115 (31.0)	Ref	
Smoking status			
Non-smoker	115 (31.0)	Ref	
Current smoker	35 (50.7)	0.815	0.196–3.393
Other tobacco use	5 (25.0)		
HIV			
Yes	91 (47.9)	0.845	0.309–2.310
No	64 (23.7)	Ref	
Haemoglobin A1c (%)			
< 6.5 (inconclusive)	117 (35.2)	Ref	
≥ 6.5 (diabetic)	33 (27.5)	1.050	0.548–2.012
Random plasma glucose (mmol/L)			
< 5.6 (diabetes excluded)	110 (40.1)	Ref	
5.6–11.0 (inconclusive)	42 (26.1)	0.907	0.495–1.661
≥ 11.1 (diabetic)	3 (12.0)	0.102	0.017–0.604*
Newly diagnosed NCD			
Yes	129 (37.9)	3.193	1.620–6.293*
No	26 (21.7)	Ref	
On diabetes medication			
Yes	147 (35.1)	1.219	0.398–3.739
No	8 (19.5)	Ref	
On antiretroviral medication			
Yes	59 (23.7)	2.037	0.818–5.069
No	96 (45.5)	Ref	
Framingham 10-year risk category			
Low risk	46 (25.7)	Ref	
Medium risk	81 (37.0)	2.683	1.474–4.881*
High risk	18 (38.3)	5.088	1.887–13.718*
Very high risk	5 (62.5)	19.909	2.253–175.902*

^#^Likelihood ratio reported because of cells with expected counts <5*cells with expected counts <5 statistically significant.

### Factors associated with referral for further care

Examining the pattern of referral for further care among the study population (Table 4), we found that individuals were more likely to be referred if they had been newly diagnosed with NCDs including diabetes, dyslipidaemia and kidney disease with impaired estimated GFR.

**Table 4:** Factors associated with referral for further care.

	Referred for further care (*n* = 453)			Chi square	*p* value
Variable	Yes	No	Total		
Sex					
Female	265 (78.6)	72 (21.4)	337 (100)	0.123	0.792
Male	93 (80.2)	23 (19.8)	116 (100)		
Age					
19 to 29	35 (70.0)	15 (30.0)	50 (100)	9.326	0.053
30 to 39	98 (76.0)	31 (24.0)	129 (100)		
40 to 49	117 (83.0)	24 (17.0)	141 (100)		
50 to 59	65 (87.8)	9 (12.2)	74 (100)		
≥ 60	43 (72.9)	16 (27.1)	59 (100)		
BMI					
Normal weight (18.5–24.9)	71 (83.5)	14 (16.5)	85 (100)	2.699	0.609
Pre-obesity (25.0–29.9)	64 (79.0)	17 (21.0)	81 (100)		
Obesity class I (30.0–34.9)	110 (79.1)	29 (20.9)	139 (100)		
Obesity class II (35.0–39.9)	54 (73.0)	20 (27.0)	74 (100)		
Obesity class III (≥ 40)	59 (79.7)	15 (20.3)	74 (100)		
Haemoglobin A1c (%)					
< 6.5 (inconclusive)	262 (79.2)	69 (20.8)	331 (100)	0.060	0.807
≥ 6.5 (diabetic)	89 (78.1)	25 (21.9)	114 (100)		
Random plasma glucose (mmol/L)					
< 5.6 (diabetes excluded)	213 (78.6)	58 (21.4)	271 (100)	2.476	0.290
5.6–11.0 (inconclusive)	123 (77.8)	35 (22.2)	158 (100)		
≥ 11.1 (diabetic)	22 (91.7)	2 (8.3)	24 (100)		
HPT					
Yes	112 (78.9)	30 (21.1)	142 (100)	0.003	1.000
No	246 (79.1)	65 (20.9)	311 (100)		
Newly diagnosed NCD					
Yes	291 (86.9)	44 (13.1)	335 (100)	47.659	<0.001*
No	67 (56.8)	51 (43.2)	118 (100)		
Newly diagnosed diabetes mellitus					
Yes	24 (100)	0 (0.0)	24 (100)	6.986^+^	0.008*
No	269 (85.9)	44 (14.1)	313 (100)		
Newly diagnosed HPT					
Yes	48 (92.3)	4 (7.7)	52 (100)	1.598	0.266
No	243 (85.9)	40 (14.1)	283 (100)		
Newly diagnosed dyslipidaemia					
Yes	114 (98.3)	2 (1.7)	116 (100)	20.369	<0.001*
No	176 (80.7)	42 (19.3)	218 (100)		
Newly diagnosed kidney disease					
Yes	146 (100)	0 (0.0)	146 (0.0)	38.905	<0.001*
No	146 (76.8)	44 (23.2)	190 (100)		
Estimated GFR category					
G1: Normal or high	214 (71.8)	84 (28.2)	298 (100)	34.596^#^	<0.001*
G2: Mildly decreased	120 (92.3)	10 (7.7)	130 (100)		
G3a: Mildly to moderately decreased	16 (100)	0 (0.0)	16 (100)		
G3b: Moderately to severely decreased	6 (85.7)	1 (14.3)	7 (100)		
G4: Severely decreased	2 (100)	0 (0.0)	2 (100)		
Estimated GFR in two categories					
Normal or high GFR (G1)	214 (71.8)	84 (28.2)	298 (100)	27.368	<0.001*
Decreased GFR (G2 to G4)	144 (92.9)	11 (7.1)	155 (100)		
Albumin-creatinine ratio					
<3 mg/mmol	345 (78.9)	92 (21.1)	437 (100)	0.161	0.689
3 – 30 mg/mmol	13 (86.7)	2 (13.3)	15 (100)		
Framingham 10-year risk category					
Low risk	132 (75.0)	44 (25.0)	176 (100)	2.934	0.403
Medium risk	177 (81.6)	40 (18.4)	217 (100)		
High risk	36 (80.0)	9 (20.0)	45 (100)		
Very high risk	7 (87.5)	1 (12.5)	8 (100)		

^#^Likelihood ratio reported because of cells with expected counts <5 *statistically significant.

## Discussion

This study serves as a pilot to demonstrate that integrating screening for NCDs including HPT, T2D, obesity, dyslipidaemia, and kidney disease, into health care services is achievable. Future studies will be needed to establish cost effectiveness and implementation.

Using existing infrastructure and staff, a nurse-led approach identified a high burden of undiagnosed NCDs among adults attending PHC clinic and HIV treatment clinic in Johannesburg.

The WHO advocates POC testing for NCDs as part of its package for essential intervention in PHC especially for LMICs (14). There are advantages and barriers to POC testing that should be considered when scalling a screening program. A review study by Tenorio-Mucha et al. (14) revealed advantages to POC such as convenience, simple tests, real time results facilitating medical decision making, as well as barriers such as accuracy of results when starting patients on treatment for life. Szablowski et al. (15) found POC HbA1c tests to be as accurate as lab based. Mutepfa et al. (16) evaluation of POC cholesterol machines can be used in PHC as long as they are validated. We opted to use POC testing due to convenience for the participants and resourse constraints and had referral systems to a medical doctor for those with abnormalities.

Our findings align with previous studies showing the utility of POC screening in PHC settings.

For example, Brennan et al. (17) integrated POC screening for HPT and T2D into South Africa’s COVID-19 vaccine programme and identified similarly high levels of undiagnosed disease. However, our study expands this approach by incorporating kidney function assessment and a broader range of cardiometabolic risk factors. Compared to Brennan et al.’s cohort, we found higher prevalence of HPT (31.3% vs. 19%) and a slightly lower rate of newly diagnosed T2D (5.9% vs. 7.8%), likely reflecting differences in demographic and clinical profiles.

Around half of the participants drank alcoholic beverages, the most common form being beer (24.1%) on a weekly basis. Alcohol consumption has been linked globally to 474000 CVD in 2019 (18). The level of risk of CVD from alcohol is linked to the volume of alcohol consumed at a time. Unfortunately, we did not measure this in the study and are unable to define if this is high risk, we can only assume that it has an effect due to the high percentage reported. To our surprise 80.7% of the participants were non-smokers. We suspect this is in keeping with patients receiving health education while taking meds for HIV and previous CVD. Almost 50 percent of the population were unemployed and from poorer communities in Johannesburg which can also be considered a risk factor for CVD.

A key secondary finding was the high proportion of participants with obesity (63.9%), particularly among women. This is consistent with national data showing higher obesity rates among South African women and aligns with global concerns about rising obesity-related cardiovascular risk (19). In 2019 five million deaths from NCDs were attributed to high BMI (20). We also observed a high burden of kidney disease (31.7%), identified using eGFR thresholds from the 2024 KDIGO CKD guidelines. Predictors of kidney impairment included female sex, older age, obesity, HIV status, family history of NCDs, and both newly diagnosed and treated diabetes. Interestingly, HPT did not emerge as a significant predictor, which may be explained by the relatively young mean age of our cohort (44.4 years), where end-organ damage may not yet be clinically apparent. End organ complications usually take years to be detectable, hence, the high prevalence of HPT in this population implies the potential for end organ damage including renal impairment a few years down the line. This is therefore a population to watch and screen at regular intervals, so changes are detected early.

The observed clustering of metabolic, renal, and cardiovascular risk factors supports the emerging concept of cardiovascular-kidney-metabolic (CKM) syndrome (21). This syndemic model recognises the shared biological pathways and social determinants that drive overlapping chronic conditions. Each of these 3 conditions can lead to or worsen the other leading to complications such as heart or kidney failure (21). Screening for this syndrome involves looking at basic biological assessments, POC tests as well social determinants of health such as education, unemployment and mental health issues (21). Monitoring BP, lipid profiles, blood glucose, BMI, and kidney function is imperative for the identification and management of CKM as the syndrome is divided into stages that determine the medical intervention.

The age profile of the cohort explains the absence of significant associations between BMI, hypertension, and kidney impairment in the multivariable model. Age was the dominant predictor of kidney impairment (AOR 4.866 for ages 45–64; AOR 19.011 for ≥65), yet the mean age was 44.4 years with 39.1% of participants under 40—below the threshold at which obesity-related nephropathy and hypertensive nephrosclerosis typically become clinically manifest. The high burden of both risk factors in this young cohort reflects a population at an early but critical stage of chronic disease progression, where timely intervention and long-term surveillance offer the greatest opportunity to prevent future CKD.

The majority of our cohort came from low-income communities, and nearly half were unemployed, factors known to increase vulnerability to NCDs through limited access to healthy food, physical activity, and healthcare.

Despite strengths, this study has several limitations. Convenience sampling used can lead to selection bias there is a potential for single time point measurement bias. The study attempted to mitigate this bias by ensuring instruments across both sites were identical, validated with suppliers has having high sensitivity and specificity and approved protocol were followed by both sites. However, we did not do multiple sample measurements or multivariate analysis which opened to this bias. Single-occasion assessments are subject to biological and measurement variability and may over- or underestimate true disease prevalence. All participants with abnormal screening results were referred to a clinician for confirmatory assessment. Prevalence estimates from this study should therefore be interpreted as screening yields rather than confirmed disease prevalence.

We were unable to distinguish between type 1 diabetes and T2D among participants already on treatment, which may have introduced misclassification. While referrals were made for participants with abnormal findings, we did not track whether they accessed follow-up care, limiting our ability to assess downstream clinical impact. Alcohol use was reported but not quantified, preventing an assessment of dose-dependent cardiovascular risk. Finally, the cross-sectional design limits causal inference.

Data in Table 2 demonstrates higher rates of newly diagnosed NCDs among HIV-positive participants, these comparisons are unadjusted and may reflect confounding by ART use, immune status, and clinical characteristics. A fully adjusted analysis of NCD outcomes stratified by HIV status is warranted in future, larger studies, and should account for ART regimen type, CD4 count, viral load, and duration of infection. Our findings have several implications for practice and policy. They support expanding routine NCD screening within PHC using existing personnel, particularly nurses, with minimal disruption to services. The high burden of undiagnosed and overlapping conditions underscores the need for integrated, person-centered care. In settings with a high prevalence of HIV and constrained health resources, CKM screening may offer a cost-effective approach to identifying individuals at risk for multiple chronic conditions. Additionally, the disproportionate burden among women highlights the importance of gender-responsive strategies.

Future research should evaluate the long-term outcomes of integrated NCD screening, including linkage to care, treatment uptake, and clinical endpoints such as cardiovascular events or renal decline. Implementation studies should also explore how best to operationalise CKM screening within PHC, including task-shifting models, digital decision support, and integration with existing HIV and maternal health programmes.

## Glossary List of commonly used abbreviationsAbbreviationDescription

ACC: American college of cardiology
AHC: American heart association
ART: Antiretroviral therapy
BP: Blood pressure
CKD: Chronic kidney disease
COVID-19: Coronavirus disease-19
Crr: Creatinine
CKM: Cardiovascular-Kidney -Metabolic
CVD: Cardiovascular disease
e-GFR: Estimated glomerular filtration rate
HbA1C: Haemoglobin A1C
HDL: High density lipoprotein
HPT: Hypertension
HREC: Human research ethics committee
ICF: Informed consent form
LMIC: Lower- and middle-income countries
NCDs: Non-communicable diseases
POC: Point of care
PHC: Primary health care
SAHPRA: South african health products regulatory authority
SEMDSA: Society of endocrine, metabolism, and diabetes of South Africa
SDG: Sustainable development goal
SA: South africa
T2D: Type 2 diabetes mellitus
TB: Tuberculosis
WHO: World health organisation
