# Supplementary material for: Integrating non-communicable disease screening to COVID-19 and human immunodeficiency virus care in Johannesburg South Africa: a pilot model for low- and middle-income countries
Source: Front Public Health. 2026 Sep 17;14:1825868. doi: 10.3389/fpubh.2026.1825868 (PMC13628492; doi:10.3389/fpubh.2026.1825868)
Supplement: Supplementary file 1 [file Data_Sheet_1.docx]

## Appendices

### Appendix 1: Non-Communicable Disease diagnostic values

Values for diagnosis and monitoring of HPT, T2D and dyslipidaemia are all based on current SA guidelines as prescribed by SA HPT society, SEMDSA and SA heart society respectively. Obesity is defined using BMI values as per WHO classification.

#### HPT

The diagnosis of HPT was based on the criteria below:

**Table 5: Definition for HPT**

| **Current Southern African Hypertension Society Definition of hypertension** | | | |
| --- | --- | --- | --- |
| ***BP category**** | ***SBP*** |  | ***DBP*** |
| Normal | < 120 | and | < 80 |
| Optimal | 120–129 | and | < 80 |
| High normal | 130–139 | or | 80–89 |
| ***HPT*** |  |  |  |
| Grade 1 | 140–159 | or | 90–99 |
| Grade 2 | 160–179 | or | 100–109 |
| Grade 3 | ≥ 180 | or | ≥ 110 |
| Isolated systolic | ≥ 140 | and | < 90 |
| *Individuals with SBP and DBP in two categories were designated to higher BP reading recorded. | | | |

Adapted from: South African Hypertension Society commentary on the American College of Cardiology /American Heart Association Hypertension guidelines.CV journal of Africa. June 2019. ^22,23,24^.

#### T2D:

For purpose of the study the diagnosis was based on:

- Random Blood glucose ≥11.1 mmol/L
- HbA1C ≥ 6.5%

The above recommendations are Adapted from SEMDSA type 2 diabetes guideline.2017.^25^

**Table 6: Test interpretation for Screening and Diagnosis of Diabetes**

| **Interpretation of Tests Used for Screening and Diagnosis of Diabetes** | | | |
| --- | --- | --- | --- |
| Fasting plasma glucose^a^  (FPG)^a^ (mmol/L) | <5.6  Diabetes excluded | 6.0 - 6.9  Impaired fasting glucose | ≥ 7.0  Diabetes |
| 2hr-plasma glucose  (2-hr PG)^b^(mmol/L) | <7.8  Normal glucose tolerance | 7.8 - 11.0  Impaired glucose tolerance | ≥ 11.1  Diabetes |
| Glycated haemoglobinA1c  (HbA1c)^c^ (%) | <6.5  Inconclusive |  | ≥ 6.5  Diabetes |
| Random plasma glucose  (RPG)^d^  (mmol/L) | <5.6  Diabetes excluded | 5.6 - 11.0  Inconclusive | ≥ 11.1  Diabetes |
| ^a^ Fasting is defined as no caloric intake for at least 8 hours.  ^b^2-h PG is measured during an oral glucose tolerance test (OGTT); The test should be performed as described by the WHO, using a glucose load containing the equivalent of 75 g anhydrous glucose dissolved in 250 ml water and ingested over five minutes.  ^c^HbA1c: refers to glycated haemoglobin measurement performed using a DCCT standardized assay and an NGSP certified laboratory, in the absence of conditions that preclude its accurate measurement.  ^d^ Random (casual) is defined as any time of day, without regard to time of last meal. | | | |

Adapted from SEMDSA type 2 diabetes guideline.2017.^25^

#### Dyslipidaemia:

Point of care testing for total cholesterol was done. If the finger-prick total cholesterol measurement is high (>5 mmol/L), patients were referred to their doctor for a full lipogram.

*Cholesterol classification*

The classification for cholesterol was based on the following guidelines for persons 20 years and older.

Table 7: Cholesterol

| Total cholesterol | Acceptable | <200mg/dl |
| --- | --- | --- |
|  | Borderline high | 200–239mg/dl |
|  | High | ≥240mg/dl |
| HDL | Acceptable | ≥60mg/dl |
|  | Borderline low | 40–59mg/dl |
|  | Risk of heart disease | <40mg/dl |

Adapted from Medical News today: What should my cholesterol be at my age: Medical News today.^26^

#### Obesity

Obesity is widely measured using BMI (measured as BMI=weight in kg/height in m^2^) and was classified according to the table below.

**Table 8: Body Mass Index values and Class:**

| **BMI** | **Class** |
| --- | --- |
| Below 18.5 | Underweight |
| 18.5–24.9 | Normal weight |
| 25.0–29.9 | Pre-obesity |
| 30.0–34.9 | Obesity class I |
| 35.0–39.9 | Obesity class II |
| Above 40 | Obesity class III |

Adapted from WHO-Europe BMI.^27^

#### CV risk score/Framingham risk score:

The Framingham risk score provides an estimate of the 10-year risk of total CVD (fatal and non-fatal coronary events, cerebrovascular and peripheral arterial disease, and heart failure).

##### Framingham risk score: Cardiovascular Risk Stratification

**Risk Scoring and Interpretation**:

The Framingham Risk table is a 10-year risk assessment chart for patients without diabetes or severe monogenic disorders (e.g., FH) indicates risk of total CVD (coronary heart disease, stroke, peripheral artery disease or heart failure).

**Table 9: Framingham Risk Assessment Table**

| **Estimate of 10-year risk of CVD for men** | | |  | | **Estimate of 10-year risk of CVD for women** | |
| --- | --- | --- | --- | --- | --- | --- |
| **Age (yrs)** | **Points** |  | | **Age (yrs)** | | **Points** |
| 30 – 34 | 0 |  |  | 30 - 34 | | 0 |
| 35 – 39 | 2 |  |  | 35 - 39 | | 2 |
| 40 – 44 | 5 |  |  | 40 - 44 | | 4 |
| 45 – 49 | 6 |  |  | 45 - 49 | | 5 |
| 50 – 54 | 8 |  |  | 50 - 54 | | 7 |
| 55 – 59 | 10 |  |  | 55 - 59 | | 8 |
| 60 – 64 | 11 |  |  | 60 - 64 | | 9 |
| 65 – 69 | 12 |  |  | 65 - 69 | | 10 |
| 70 – 74 | 14 |  |  | 70 - 74 | | 11 |
| 75 years or older | 15 |  |  | 75 years or older | | 12 |
|  | | |  | |  | |
| **Total cholesterol (mmol/l)** | **Points** |  | | **Total cholesterol (mmol/l)** | | **Points** |
| <4.10 | 0 |  |  | <4.10 | | 0 |
| 4.10 - 5.19 | 1 |  |  | 4.10 - 5.19 | | 1 |
| 5.20 - 6.19 | 2 |  |  | 5.20 - 6.19 | | 3 |
| 6.20 - 7.20 | 3 |  |  | 6.20 - 7.20 | | 4 |
| >7.20 | 4 |  |  | >7.20 | | 5 |
|  | | |  | |  | |
| **HDL-cholesterol (mmol/l)** | **Points** |  | | **HDL-cholesterol (mmol/l)** | | **Points** |
| ≥1.50 | -2 |  |  | ≥1.50 | | -2 |
| 1.30 - 1.49 | -1 |  |  | 1.30 - 1.49 | | -1 |
| 1.20 - 1.29 | 0 |  |  | 1.20 - 1.29 | | 0 |
| 0.90 - 1.19 | 1 |  |  | 0.90 - 1.19 | | 1 |
| <0.90 | 2 |  |  | <0.90 | | 2 |
|  | | |  | |  | |
| **Systolic BP – untreated (mmHg)** | **Points** |  | | **Systolic BP – untreated (mmHg)** | | **Points** |
| <120 | -2 |  |  | <120 | | -3 |
| 120 – 129 | 0 |  |  | 120 - 129 | | 0 |
| 130 - 139 | 1 |  |  | 130 - 139 | | 1 |
| 140 - 159 | 2 |  |  | 140 - 149 | | 2 |
| ≥160 | 3 |  |  | 150 - 159 | | 4 |
|  |  |  |  | ≥160 | | 5 |
|  | | |  | |  | |
| **Systolic BP – on antihypertensive treatment (mmHg)** | **Points** |  | | **Systolic BP – on antihypertensive treatment (mmHg)** | | **Points** |
| <120 | 0 |  |  | <120 | | -1 |
| 120 – 129 | 2 |  |  | 120 - 129 | | 2 |
| 130 – 139 | 3 |  |  | 130 - 139 | | 3 |
| 140 - 159 | 4 |  |  | 140 - 149 | | 5 |
| ≥160 | 5 |  |  | 150 - 159 | | 6 |
|  |  |  |  | ≥160 | | 7 |
|  | | |  | |  | |
| **Smoker** | **Points** |  | | **Smoker** | | **Points** |
| No | 0 |  |  | No | | 0 |
| Yes | 4 |  |  | Yes | | 3 |

| **Points total for men** | |  | **Points Total for women** | |
| --- | --- | --- | --- | --- |
| **Points total** | **10-year risk (%)** |  | **Points total** | **10-year risk (%)** |
| -3 or less | <1 |  | -2 or less | <1% |
| -2 | 1.1 |  | -1 | 1 |
| -1 | 1.4 |  | 0 | 1.1 |
| 0 | 1.6 |  | 1 | 1.5 |
| 1 | 1.9 |  | 2 | 1.8 |
| 2 | 2.3 |  | 3 | 2.1 |
| 3 | 2.8 |  | 4 | 2.5 |
| 4 | 3.3 |  | 5 | 2.9 |
| 5 | 3.9 |  | 6 | 3.4 |
| 6 | 4.7 |  | 7 | 3.9 |
| 7 | 5.6 |  | 8 | 4.6 |
| 8 | 6.7 |  | 9 | 5.4 |
| 9 | 7.9 |  | 10 | 6.3 |
| 10 | 9.4 |  | 11 | 7.4 |
| 11 | 11.2 |  | 12 | 8.6 |
| 12 | 13.2 |  | 13 | 10 |
| 13 | 15.6 |  | 14 | 11.6 |
| 14 | 18.4 |  | 15 | 13.5 |
| 15 | 21.6 |  | 16 | 15.6 |
| 16 | 25.3 |  | 17 | 18.1 |
| 17 | 29.4 |  | 18 | 20.9 |
| 18 or more | >30 |  | 19 | 24 |
|  | |  | 20 | 27.5 |
| *Point totals indicate the 10-year risk of cardiovascular disease (coronary, cerebrovascular and peripheral arterial disease, and heart failure). \| Low Risk \| \| Medium Risk \| \| High Risk \| \| Very High Risk | | | | |

Adapted from Mosca L, et al., Effectiveness-based guidelines for the prevention of cardiovascular disease in women 2011 update: A guideline from the American Heart Association. Circulation 2011;123:1243-1262 ^28^ and D’Agostino RB, et al., General cardiovascular risk profile for use in primary care: the Framingham Heart Study. Circulation 2008;117:743-753.^29^

#### Kidney disease classification (using the KDIGO-2024 CKD guideline)

##### **Table 10: Classification by GFR**

| **GFR category** | **GFR (ml/min per 1.73m^2^)** | **Terms** |
| --- | --- | --- |
| G1 | ≥90 | Normal or high |
| G2 | 60 – 89 | Mildly decreased |
| G3a | 45 – 59 | Mildly to moderately decreased |
| G3b | 30 – 44 | Moderately to severely decreased |
| G4 | 15 – 29 | Severely decreased |
| G5 | <15 | Kidney failure |

##### **Table 11: Microalbuminuria classification**

| **Albuminuria category** | **Albumin creatinine ratio (mg/g)** | **Terms** |
| --- | --- | --- |
| A1 | <3mg/mmol | Normal to mildly increased |
| A2 | 3–30 mg/mmol | Moderately increased |
| A3 | >30 mg/mmol | Severely increased |

Adapted from: Improving Global Outcomes (KDIGO) CKD Work Group. 2012. Clinical Practice Guideline for the Evaluation and Management of Chronic Kidney Disease.^30^

**References:**

22. Southern African Hypertension Society. South African hypertension practice guideline 2019. Johannesburg: Southern African Hypertension Society (2019). Available online at: https://www.hypertension.org.za/guidelines (Accessed December 25, 2025).

23. Seedat YK, Rayner BL, Veriava Y, Hypertension Guideline Working Group. South

African hypertension practice guideline 2014. Cardiovasc J Afr. (2014) 25:288–94. doi:

10.5830/CVJA-2014-062.

24. Whelton PK, Carey RM, Aronow WS, Casey DE Jr, Collins KJ, Dennison Himmelfarb C, et al. 2017 ACC/AHA/AAPA/ABC/ACPM/AGS/APhA/ASH/ASPC/NMA/PCNA guideline for the prevention, detection, evaluation, and management of high blood pressure in adults: a report of the American College of Cardiology/American Heart Association Task Force on clinical practice guidelines. Hypertension. (2018) 71:1269–1324. doi:10.1161/CIR.0000000000000596.

25. Society for Endocrinology, Metabolism and Diabetes of South Africa (SEMDSA). SEMDSA 2017 guidelines for the management of type 2 diabetes mellitus. Johannesburg: SEMDSA (2017). Available online at: http://www.kznhealth.gov.za/family/SEMDS-2017-Guidelines (Accessed December 30, 2025).

26. Medical News Today. What should my cholesterol level be at my age? Medical News Today (2021). Available online at: https://www.medicalnewstoday.com/articles/315900 (Accessed December 25, 2025).

27. World Health Organization. A healthy lifestyle – WHO recommendations. Copenhagen: WHO Regional Office for Europe (2023). Available online at: https://www.who.int/europe/news-room/fact-sheets/item/a-healthy-lifestyle---who-recommendations (Accessed December 20, 2025).

28. Mosca L, Benjamin EJ, Berra K, Bezanson JL, Dolor RJ, Lloyd-Jones DM, et al.

Effectiveness-based guidelines for the prevention of cardiovascular disease in women

2011 update: A guideline from the American Heart Association. Circulation. (2011)

123:1243–62. doi: 10.1161/CIR.0b013e31820faaf8.

29. D’Agostino RB, Vasan RS, Pencina MJ, Wolf PA, Cobain M, Massaro JM, et al. General

cardiovascular risk profile for use in primary care: the Framingham Heart Study.

Circulation. (2008) 117:743–53. doi: 10.1161/CIRCULATIONAHA.107.699579.

30. Kidney Disease: Improving Global Outcomes (KDIGO) CKD Work Group. KDIGO 2024 clinical practice guideline for the evaluation and management of chronic kidney disease. Kidney Int. (2024) 105(Suppl 4S):S117–S314. doi:10.1016/j.kint.2023.10.018.
